# Physical exercise promotes white matter repair after ischemic stroke

**DOI:** 10.4103/NRR.NRR-D-24-00861

**Published:** 2025-04-29

**Authors:** Yating Mu, Xiaofeng Yang, Yifeng Feng, Liying Zhang, Jinghui Xu, Mingyue Li, Rui Wu, Shiying Li, Xiaofei He, Zejie Zuo, Xiquan Hu

**Affiliations:** Department of Rehabilitation Medicine, the Third Affiliated Hospital, Sun Yat-sen University, Guangzhou, Guangdong Province, China

**Keywords:** CXCL12, ischemic stroke, microglia, neuroinflammation, osteopontin, phagocytosis, physical exercise, transient middle cerebral artery occlusion, Treg cells, white matter injury

## Abstract

White matter injury is a key factor impacting stroke recovery. Physical exercise can promote white matter repair. Immune cells, especially regulatory T (Treg) cells, contribute to strengthening white matter integrity, yet little is known about the underlying mechanism. To examine this, we established a transient middle cerebral artery occlusion male mouse model. We found that physical exercise elevated brain Treg cells, thereby enhancing neurological recovery, reducing neuroinflammation, promoting myelin debris clearance, and accelerating white matter repair. Depletion of Treg cells caused a decrease in these positive effects of physical exercise. Mechanistically, the rise in osteopontin triggered by physical exercise is dampened when Treg cells are depleted. In addition, Treg-conditioned medium reduced oxygen–glucose deprivation/re-oxygenation-induced microglial inflammation and enhanced phagocytosis, which could be blocked by osteopontin antibodies. Importantly, although Treg infusion could mimic the protective effects of physical exercise, osteopontin blockade partially countered the effects of physical exercise and Treg cells. Finally, our sequencing data revealed a marked upregulation of C–X–C motif chemokine ligand 12 (CXCL12) mRNA expression subsequent to physical exercise, which was confirmed at the protein level. Stimulation of Treg cells with stroke brain lysates increased C–X–C motif chemokine receptor 4 (CXCR4) expression, indicating a potential role for the CXCL12–CXCR4 axis in recruiting Treg cells. These findings suggest that physical exercise promotes white matter repair after ischemic stroke by Treg cells.

## Graphical abstract

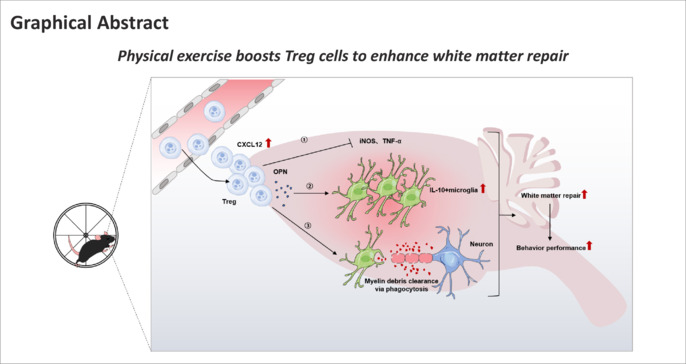

## Introduction

Stroke remains a leading cause of death and disability (Mendelson and Prabhakaran, 2021), accounting for 143 million disability-adjusted life years (GBD 2019 Stroke Collaborators, 2021) and posing a severe threat to human health. Furthermore, heightened exposure to risk factors like hyperglycemia, hypertension, and inadequate physical activity is driving a trend towards younger stroke incidence (Cabral et al., 2017). Consequently, stroke research is a top priority. Neuroprotectants administered to humans are not as successful as in preclinical trials, with one possible reason being the lack of white matter protection (Ho et al., 2005). Indeed, about 95% of ischemic stroke involves white matter injury (Ho et al., 2005). The cognitive, emotional, and sensorimotor impairments and urinary incontinence that often follow a stroke are closely associated with disruptions and remodeling of white matter connectivity (Wang et al., 2016). Therefore, white matter imaging parameters are emerging as independent predictors of stroke recovery (Sagnier et al., 2020). In addition, white matter integrity is linked to functional progression after diverse interventions, including physical exercise (PE) (Ando et al., 2020; Xu et al., 2023).

Microglia play a double-edged sword effect in maintaining the integrity of white matter. Activated M1 microglia release pro-inflammatory mediators and produce reactive oxygen species to induce white matter injury (Hartung, 1993; Chen et al., 2020b). In contrast, M2 microglia clear myelin debris via phagocytosis and the secretion of neurotrophic factors, providing an appropriate microenvironment for white matter repair (Lampron et al., 2015; Zheng et al., 2022). The immune microenvironment and intercellular crosstalk regulate microglial polarization (Liesz et al., 2009; Ricci and Liesz, 2023). Thus, modulating the microglia phenotype through alterations in the microenvironment or cell interactions is a potential therapeutic approach for improving white matter integrity.

Accumulating evidence supports the benefits of regulatory T (Treg) cells in response to ischemic stroke because of their powerful anti-inflammatory properties and repair-promoting effects (Ito et al., 2019; Shi et al., 2021; Yshii et al., 2022). Depletion of Treg cells exacerbates, while boosting them mitigates, ischemic injury and dysfunction (Liesz et al., 2009; Yshii et al., 2022). In addition, Treg cells can reprogram the microglia transcriptome, thereby shifting them towards a tissue repair phenotype (Shi et al., 2021). This is why Treg cells have a profound impact despite their relatively low abundance in the brain.

PE is widely used in clinical practice to improve neurological function in stroke survivors, with the effect partly due to its ability to strengthen white matter (Hung et al., 2022; Xu et al., 2023). PE exerts a profound effect on Treg cells. β2-Adrenergic receptors on Treg cells are stimulated by adrenaline induced during PE, leading to an increase of intracellular cAMP levels, which thereby directly enhances their suppressive capacity (Bodor et al., 2012; Dugger et al., 2018). Furthermore, exercise-inducible metabolites, such as lactate, can promote the proliferation of Treg cells (Watson et al., 2021). However, the underlying mechanisms are relatively complex and still need to be clarified. In this study, we hypothesized that Treg cells are involved in PE-induced white matter repair. We sought to determine whether Treg cells are crucial in white matter repair induced by PE post-stroke, and if so, what the underlying mechanisms might be.

## Methods

### Animals

A total of 167 C57BL/6 male mice (8 weeks, 23–26 g) purchased from GemPharmatech Co., Ltd. (Nanjing, China, license No. SCXK [Yue] 2020-0054) were used for *in vivo* experiments. The mice were housed in a dedicated specific pathogen-free grade animal facility with the temperature maintained at a constant 22 ± 2°C and relative humidity at 50% ± 10%. The lighting cycle was set to 12 hours of light and 12 hours of darkness. The mice were provided with ample food and water, and each cage housed a maximum of five mice.

The study was divided into three experiments. Experiment 1 involved three groups: Sham-operated (Sham), transient middle cerebral artery occlusion (tMCAO), and tMCAO + PE. Experiment 2 comprised two groups: PE + IgG and PE + Anti-CD25. Experiment 3 consisted of four groups: PE + IgG, PE + Anti-osteopontin (OPN), Treg + IgG, and Treg + Anti-OPN groups. Cerebral infarction was induced by tMCAO for 60 minutes, as previously described (Chiang et al., 2011). The Sham group received anesthesia (1.5% pentobarbital sodium [Sigma–Aldrich, St. Louis, MO, USA], intraperitoneally administered at 0.005 mL/g body weight) and artery exposure without occlusion. The success of the model was confirmed by magnetic resonance imaging at 24 hours after tMCAO.

All experiments were carried out in accordance with the National Institutes of Health Guide for the Care and Use of Laboratory Animals and were approved by the Laboratory Animal Ethics Committee of Lai’an Scientific Co., Ltd. (Guangzhou, China; approval No. G2023023; dated April 6, 2023).

### Physical exercise

Mice assigned to the tMCAO + PE, PE + IgG, PE + Anti-CD25, and PE + Anti-OPN groups were trained using an independent running wheel (Weibi, Shanghai, China). The mice started PE at 48 hours after tMCAO, as previously recommended (Zhang et al., 2020a). During the first training week, the mice were placed in the independent running wheel (fully enclosed, 13 cm × 8 cm × 14 cm) for 30 minutes, once a day. For the following 2 weeks, the training time was increased to 1 hour.

### Behavioral tests

A series of tests that have been proven to be highly sensitive and powerful in rodent models were used to examine motor and cognitive function.

The adhesive removal test was used to examine sensorimotor function (Shi et al., 2021). Adhesive tape (2 mm × 3 mm) was pasted on the injured forepaw. The time taken to remove the tape was recorded, with an observation time of 2 minutes.

The rotarod test was used to examine muscular endurance and balance capacity (Shan et al., 2023). The mice were forced to run on a rotating rod with an initial speed of 4 r/min, accelerating to 40 r/min within 300 seconds. The latency to fall off the rotating rod was recorded. Each mouse underwent three consecutive trials with an interval of 15 minutes, and the data were presented as mean values from three trials.

The novel object recognition (NOR) test and Morris water maze (MWM) test were used to examine cognitive function (Othman et al., 2022). The NOR test was performed as previously described (Jiang et al., 2017). Briefly, mice were placed in a 40 cm × 40 cm × 40 cm open-field box with two identical objects for 5 minutes. After an interval of 1 hour, one of the objects was removed and replaced by a novel object. The mice were then placed back in the box for 5 minutes and the exploration time for both objects was recorded. The discrimination ratio was calculated as: (time to explore the novel object)/(total time to explore both objects) × 100%.

The MWM Experimental Analysis System for Rats and Mice test (Shanghai Xinruan Information Technology Co., Ltd., Shanghai, China) was conducted in a large circular pool (diameter: 120 cm, height: 76 cm) filled with 35 cm of water. The MWM test included hidden platform trials and a spatial probe trial. In the hidden platform trials, a 10-cm platform was submerged approximately 1.0 cm below the surface of the water and placed in the center of any quadrant. The mice were placed in the water facing the pool wall in one of the four quadrants and allowed to locate the platform within 60 seconds. If unsuccessful within the allotted time, the mice were physically guided to the platform and allowed to stay there for 30 seconds. Each mouse underwent four trials per day, with the daily order of entry into the quadrants randomized. The time taken to reach the platform was recorded to reflect learning ability. In the spatial probe trial, the platform was removed, but the observation time was still 60 seconds. The time spent in the goal quadrant and the number of times crossing the former platform position were recorded to reflect memory ability.

All behavioral tests were carried out following a 21-day intervention period.

### Histological staining

Following a 21-day intervention, the mice were anesthetized (1.5% pentobarbital sodium, 0.02 mL/g) and perfused with cold saline, followed by 4% paraformaldehyde in phosphate buffered saline. Brains were subjected to gradient dehydration in 20% and 30% sucrose solutions at 4°C. A freezing microtome (Leica, Wetzlar, Germany) was used to cut coronal brain sections at 10 or 30 μm thickness.

For Luxol fast blue staining, sections were immersed in 0.1% Luxol fast blue (Sigma–Aldrich) solution at 60°C for 20 minutes and then sequentially immersed in 95% alcohol, distilled water, 0.05% lithium carbonate, and 70% alcohol to remove excess staining until the white matter could be clearly distinguished. Images of the peri-infarct areas were captured.

For immunofluorescence staining, sections were blocked with 1% bovine serum albumin (Biotopped, Beijing, China) in 0.3% Triton X-100 in phosphate buffered saline for 60 minutes, followed by incubation with primary antibodies at 4°C overnight. After rinsing three times with phosphate buffered saline for 10 minutes, sections were incubated with secondary antibodies for 60 minutes at 26°C. The primary antibodies used in this study were: anti-myelin basic protein (MBP; rabbit pAb, 1:100, Servicebio, Wuhan, China, Cat# GB11226, RRID: AB_2895014), anti-ionized calcium-binding adapter molecule 1 (Iba1; mouse mAb, 1:100, Servicebio, Cat# GB12105, RRID: AB_2922434), anti-Iba1 (rabbit mAb, 1:500, Wako, Cat# 019-19741, Japan, RRID: AB_839504), anti-interleukin 10 (IL-10; rabbit mAb (1:100, Abclonal, Wuhan, China, Cat# A12255, RRID: AB_3665480), and anti-degraded myelin basic protein (dMBP; rabbit pAb, 1:500, Millipore, Bedford, MA, USA, Cat# AB5864, RRID: AB_2140351). The secondary antibodies used in this study were: Alexa Fluor® 568 goat anti-mouse IgG (1:500, Abcam, Cambridge, UK, Cat# ab175473, RRID: AB_2895153), Alexa Fluor® 488 goat anti-rabbit IgG (1:500, CST, Danfoss, MA, USA, Cat# 4412S, RRID: AB_1904025), and Alexa Fluor® 555 goat anti-rabbit IgG (1:500, CST, Cat# 4413S, RRID:AB_10694110). Images were obtained using a fluorescence microscope (Eclipse Ni-U; Nikon, Tokyo, Japan). Image analyses were conducted on one or two randomly selected fields within the peri-infarct regions of each section. The average positive cell count of two sections was analyzed for each mouse brain.

Transmission electron microscopy was used to examine the myelin sheath microstructure in the external capsule, consistent with the method used in our previous study (He et al., 2024).

### Flow cytometry

After 3, 7, 14, and 21 days, the mice were anesthetized and perfused with cold saline. The ipsilateral hemispheres were collected and brain homogenates were prepared using a glass homogenizer (Sigma–Aldrich). Homogenates were then passed through a 70-μm cell strainer (BD, Franklin Lakes, NJ, USA). Samples were centrifuged at 300 × *g* for 5 minutes at 4°C. The pellet was resuspended in 10 mL of 40% Percoll. Samples were centrifuged at 500 × *g* for 30 minutes at 4°C with maximal acceleration and deceleration. The cells were washed with 1× Hank’s balanced salt solution. The single-cell suspension was incubated with antibodies to surface antigens: CD4-APC (eBioscience, San Diego, CA, USA, Cat# 17-0042-82, RRID:AB_469323) and CD25-eFluor^TM^ 450 (eBioscience, Cat# 48-0251-82, RRID:AB_10671550) for 40 minutes at 4°C in the dark. After washing, the cells were fixed and permeabilized using Fixation/Permeabilization buffer (Thermo Fisher Scientific, Waltham, MA, USA) according to the manufacturer’s protocol. Without washing, the forkhead box P3 (Foxp3) antibody (eBioscience, Cat# 12-5773-82, RRID:AB_465936) was added to the cells and incubated for 40 minutes at room temperature in the dark. Flow cytometry was performed on a flow cytometer (Beckman Coulter, Brea, CA, USA), and data were analyzed using FlowJo software (Treestar, San Carlos, CA, USA).

### Treg cell depletion

To deplete Treg cells, the mice in the PE + Anti-CD25 group were intraperitoneally injected with CD25-specific antibody (clone PC61, 102058, Biolegend, San Diego, CA, USA). The CD25 antibody was administered 24 hours before PE at a dose of 250 μg per mouse, and then injected once a week for two further administrations. The mice in the PE + IgG group received an isotype IgG control (normal rat IgG control, 6-001-F, Biolegend).

### Osteopontin blockade

The mice in the PE + Anti-OPN and Treg + Anti-OPN groups were intracerebroventricularly injected with OPN antibody (AF808, R&D Systems, Minneapolis, MN, USA) to block OPN. The OPN antibody was administered 24 hours before PE at a dose of 1 μg per mouse, and then injected once a week for two further administrations. The mice in the PE + IgG and Treg + IgG groups received an isotype IgG control (normal goat IgG control, AB-108-C, R&D Systems).

### Western blot assay

Protein was extracted from peri-infarct brain tissue via sonication, using radio immunoprecipitation assay lysis buffer containing 1% phenylmethanesulfonyl fluoride. Protein concentration was determined using a bicinchoninic acid assay kit (Beyotime, Shanghai, China). A sodium dodecyl sulfate–polyacrylamide gel electrophoresis system (Bio-Rad, Hercules, CA, USA) was used to separate proteins. The proteins were then transferred onto polyvinylidene fluoride membrane. The membrane was blocked with 5% skimmed milk at room temperature for 60 minutes and incubated with primary antibodies overnight at 4°C. After rinsing the membrane three times in Tris-buffered saline with Tween-20 for 5 minutes, the membrane was incubated with secondary antibodies for 60 minutes at room temperature. The expression of specific proteins was detected using an Enhanced Pico Light Chemiluminescence Kit (EpiZyme, Shanghai, China) and analyzed by ImageJ 1.53a. The primary antibodies used in this study were: anti-inducible isoform of nitric oxide synthase (iNOS; rabbit mAb, 1:1000, Abclonal, Cat# A3774, RRID: AB_3094627), anti-tumor necrosis factor-alpha (TNF-α; rabbit pAb, 1:1000, Abclonal, Cat# A23264, RRID: AB_3665498), anti-transforming growth factor-beta (TGF-β; mouse mAb, 1:1000, Affinity, Liyang, China, Cat# BF8012, RRID: AB_3665500), anti-IL-10 (rabbit mAb, 1:1000, Abclonal, Cat# A12255, RRID: AB_3665480), anti-β-tubulin (mouse mAb, 1:1000, Affinity, Cat# T0023, RRID: AB_2813772), anti-OPN (mouse mAb, 1:1000, Abmart, Shanghai, China, Cat# MS20111, RRID: AB_3665504), and anti-C-X-C motif chemokine ligand 12 (CXCL12; rabbit Ab, 1:1000, Abmart, Cat# PK12227, RRID: AB_3665506). The secondary antibodies used in this study were: HRP goat anti-rabbit IgG (1:2000, Abclonal, Cat# AS014, RRID: AB_2769854) and HRP goat anti-mouse IgG (1:2000, Abclonal, Cat# AS003, RRID: AB_2769851). All samples were collected at 21 days after the intervention.

### Quantitative polymerase chain reaction

The RNA-Quick Purification Kit (RN001, Vazyme, Nanjing, China) was used to extract total RNA from tissue surrounding the infarct area. The concentration and purity of total RNA were measured using a NanoDrop 2000 Spectrophotometer (Thermo Fisher Scientific). Subsequently, total RNA was reverse-transcribed into cDNA using the HiScript II 1^st^ Strand cDNA Synthesis Kit (R212-01, Vazyme). Quantitative polymerase chain reaction (qPCR) was performed using the ChamQ Universal SYBR qPCR Master Mix (Q711-02, Vazyme). To ensure consistency and reliability, mRNA expression levels were normalized to endogenous glyceraldehyde-3-phosphate dehydrogenase (GAPDH). The primer pairs used are listed in **Additional Table 1**. All samples were collected at 21 days post-intervention, with the exception of the cell samples.

**Additional Table 1:** Primer sequences

Gene	Sequence (5ʹ-3ʹ)
*GAPDH*	Forward: CATCACTGCCACCCAGAAGACTG Reverse: ATGCCAGTGAGCTTCCCGTTCAG
*iNOS*	Forward: GAGACAGGGAAGTCTGAAGCAC Reverse: CCAGCAGTAGTTGCTCCTCTTC
*TNF-α*	Forward: GGTGCCTATGTCTCAGCCTCTT Reverse: GCCATAGAACTGATGAGAGGGAG
*IL-10*	Forward: CGGGAAGACAATAACTGCACCC Reverse: CGGTTAGCAGTATGTTGTCCAGC
*TGF-β*	Forward: TGATACGCCTGAGTGGCTGTCT Reverse: CACAAGAGCAGTGAGCGCTGAA
*CD68*	Forward: GGCGGTGGAATACAATGTGTCC Reverse: AGCAGGTCAAGGTGAACAGCTG
*Trem2*	Forward: CTACCAGTGTCAGAGTCTCCGA Reverse: CCTCGAAACTCGATGACTCCTC
*Spp1*	Forward: GCTTGGCTTATGGACTGAGGTC Reverse: CCTTAGACTCACCGCTCTTCATG
*CXCR4*	Forward: GACTGGCATAGTCGGCAATGGA Reverse: CAAAGAGGAGGTCAGCCACTGA

CXCR4: C-X-C chemokine receptor 4; GAPDH: glyceraldehyde-3-phosphate dehydrogenase; IL-10: interleukin 10; iNOS: inducible isoform of nitric oxide synthase; Spp1: the encoding gene for OPN; TGF-β: transforming growth factor-beta; TNF-α: tumor necrosis factor-alpha; Trem2: triggering receptor expressed on myeloid cells 2.

### Treg cell isolation, adoptive transfer, and conditioned medium

Spleens were collected from uninjured mice and then crushed using the flat end of a plunger to release splenocytes. Debris and aggregates were removed by filtering the cell suspension through a 70-μm cell strainer. Subsequently, CD4^+^CD25^+^ Treg cells were isolated using the Mouse CD4^+^CD25^+^ Regulatory T Cell Isolation Kit II (18783, STEMCELL Technologies, Vancouver, Canada).

For *in vivo* experiments, freshly isolated Treg cells (1 × 10^6^) were intracerebroventricularly injected into mice in the Treg + IgG and Treg + Anti-OPN groups.

For *in vitro* studies, Treg cells were cultured for 24 hours in Treg culture media (RPMI-1640, Gibco, Carlsbad, CA, USA) supplemented with 10% fetal bovine serum (ScienCell, Santiago, CA, USA), 1% penicillin/streptomycin, and 0.1% β-mercaptoethanol. The media also contained anti-CD3 (5 μg/mL), anti-CD28 (5 μg/mL), and IL-2 (20 ng/mL). Following this, the cells were stimulated with infarcted brain lysate (200 μg/mL) for 1 day, and the supernatants were collected as Treg-conditioned medium (Treg-CM). To prepare infarcted brain lysate, tissue surrounding the infarction was collected, processed into a suspension with an ultrasonic disintegrator, centrifuged, and the supernatant extracted.

### Cell culture and oxygen–glucose deprivation/reperfusion

BV2 microglial cells (Procell, Wuhan, China, CL0493, RRID: CVCL_0182) and MO3.13 oligodendrocyte cells (EK-Bioscience, shanghai, China, CC-Y1772, RRID: CVCL_D357) were cultured in Dulbecco’s modified Eagle medium (Gibco) containing 10% fetal bovine serum (ScienCell) at 37°C in a 5% CO_2_ incubator.

After routine cultivation for 24 hours, all cells were washed with fresh medium twice. Then the culture medium was replaced with glucose-free Dulbecco’s modified Eagle medium and the cells were cultured under ischemic conditions (1% O_2_, 94% N_2_, and 5% CO_2_) at 37°C for 3 hours. For reperfusion, the medium was replaced by fresh normal medium and maintained in an incubator under 95% air/5% CO_2_ for 24 hours. As for BV2 cells, 10% Treg-CM was added at OGD3h/R6h, and the final culture media were collected as Treg-BV2-CM. The culture media without Treg-CM from BV2 cells at OGD3h/R24h were collected as BV2-CM.

### *In vitro* phagocytosis

Fluorescent microbeads (diameter: 1 μm, L4655, Sigma–Aldrich) were incubated with BV2 cells for 60 minutes at 37°C. Following incubation, the microbeads were removed, and the cells washed three times with phosphate buffered saline. Subsequently, the cells were fixed with paraformaldehyde. The immunofluorescence staining steps were performed as previously described.

### RNA sequencing

After 3 weeks of intervention, total RNA was extracted from the peri-infarct area using TRIzol (Thermo Fisher Scientific). RNA purity was determined using a NanoDrop and RNA concentration was measured using the Qubit RNA BR (Broad-Range) Assay Kit (Thermo Fisher Scientific). RNA integrity was examined using the RNA ScreenTape assay. A mRNA library was prepared using the NEBNext® Ultra^TM^ II mRNA library preparation kit, and sequenced on an lllumina NovaSeq 6000 Sequencing System (Illumina, San Diego, CA, USA). All procedures were performed by Shanghai Novelbio Ltd. (Shanghai, China). Gene Ontology (GO) enrichment analysis was conducted using the DAVID online database (https://david.ncifcrf.gov; Huang da et al., 2009; Sherman et al., 2022). Visualization of the results was performed using the Wei Sheng Xin platform (https://www.bioinformatics.com.cn; Tang et al., 2023). Genes with |log2(FoldChange)| > 1 and adjusted *P* value < 0.05 were deemed differentially expressed.

### 3-(4,5-Dimethylthiazolyl-2)-2,5-diphenyltetrazolium bromide assay

MO3.13 cells were seeded into 96-well plates at a density of 1 × 10^4^ cells per well and subjected to the corresponding interventions. The original medium was discarded and 100 μL of fresh medium added. Then, 50 μL of 3-(4,5-dimethylthiazolyl-2)-2,5-diphenyltetrazolium bromide (MTT; E-CK-A341-1000T, Elabscience, Wuhan, China) solution was added to each well, followed by incubation in a cell culture incubator for 3 hours. The liquid in the wells was discarded and 150 μL of DMSO was added until all the purple crystals were dissolved. Absorbance at 570 nm was detected using a multi-functional microplate reader (Biotek, Burlington, VT, USA).

### Statistical analysis

Statistical analyses were conducted using GraphPad Prism (version 8.0.2 for Windows, GraphPad Software, Boston, MA, USA, www.graphpad.com). All results are presented as mean ± standard deviation (SD). Unpaired *t*-tests were used for comparisons between two groups. For comparisons among three or more groups, one-way analysis of variance followed by Tukey’s honestly significant difference test for *post hoc* comparisons was used. For repeatedly measured data, repeated measures analysis of variance was used, followed by Tukey’s honestly significant difference for *post hoc* comparisons. *P* < 0.05 was regarded as statistically significant.

## Results

### Physical exercise promotes the recovery of neurological function post-stroke

**Figure 1:**
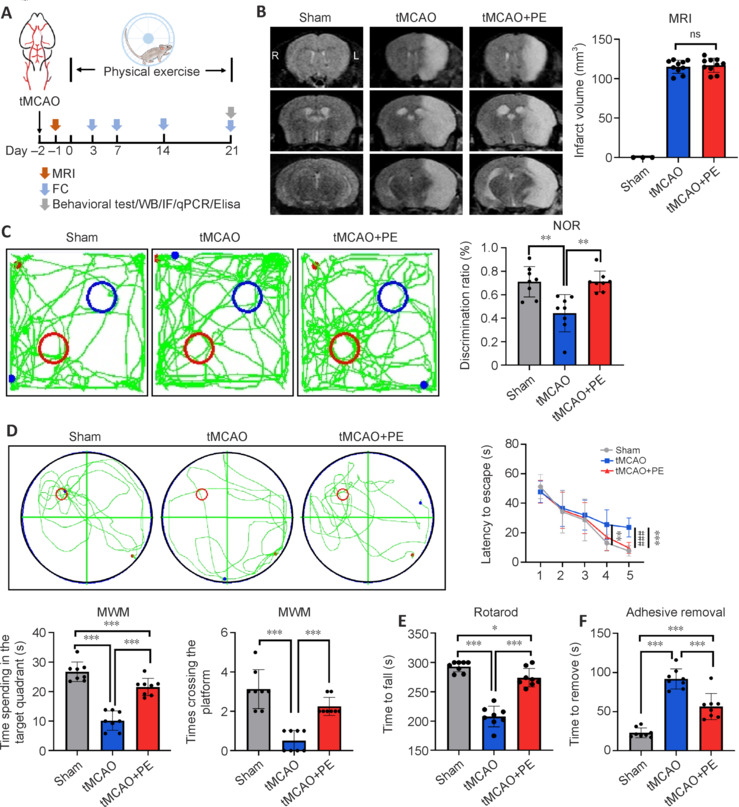
Physical exercise improves neurological function after ischemic stroke. (A) Experimental design. Created with Procreate 5.3.10. (B) T2WI at 24 hours after tMCAO showed no significant difference in infarct volume between the tMCAO and tMCAO + PE groups (*n* = 3–10). (C) The tMCAO + PE group showed superior recognition of novel objects in the NOR test. (D) The tMCAO + PE group spent a longer duration in the target quadrant in the MWM test. (E, F) Sensorimotor functions were examined using the rotarod test and adhesive test 21 days after intervention (*n* = 8). Data are expressed as mean ± SD. **P < 0.01, ***P < 0.001 (one-way analysis of variance was used for bar charts; whereas repeated measures analysis of variance was used for line charts). FC: Flow cytometry; IF: immunofluorescence; MRI: magnetic resonance imaging; MWM: Morris water maze test; NOR: novel object recognition test; PE: physical exercise; Sham: sham-operated; tMCAO: transient middle cerebral artery occlusion; WB: Western blot.

To determine the potential effects of PE on brain ischemic injury, animals were randomly assigned to three groups: Sham, tMCAO, and tMCAO + PE (**Figure 1A**). Magnetic resonance imaging was conducted 24 hours post-stroke to confirm successful establishment of the model. T2-weighted imaging revealed no significant difference in infarct volumes between the tMCAO and tMCAO + PE groups (*P* > 0.05; **Figure 1B**). Cognitive function was examined using the NOR and MWM tests, and had improved in the tMCAO + PE group compared with the tMCAO group. Specifically, mice in the tMCAO + PE group showed a higher discrimination ratio in the NOR test (*P* < 0.01; **Figure 1C**). During the spatial acquisition phase of the MWM test, mice in the tMCAO + PE group exhibited shorter latencies to find the hidden platform, indicating superior learning skills. In the probe trial, mice in the tMCAO + PE group spent more time in the target quadrant and crossed the platform more frequently than those in the tMCAO group (**Figure 1D**). Furthermore, PE enhanced post-stroke sensorimotor functions, as shown by increased time spent on the rotating bar in the rotarod test (*P* < 0.01; **Figure 1E**), and reduced time for removal of adhesive tape from injured paws in the adhesive removal test (*P* < 0.01; **Figure 1F**). However, mice in the tMCAO + PE group did not fully recover to the same level as mice in the Sham group.

### Physical exercise enhances white matter repair after ischemic stroke

**Figure 2:**
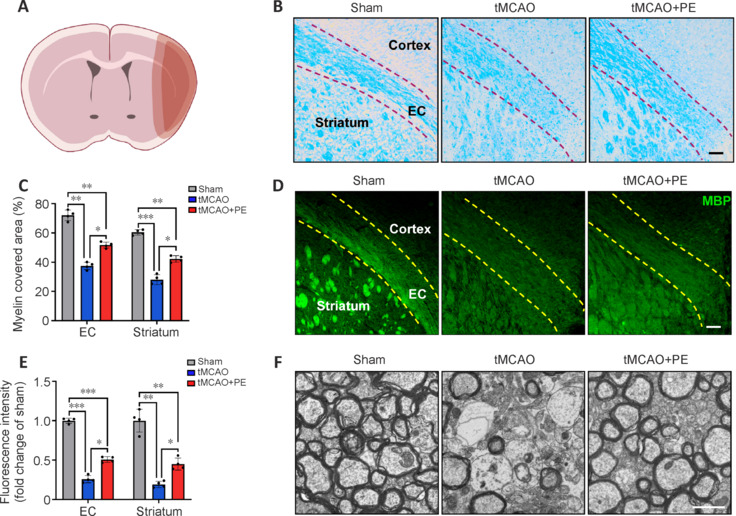
Physical exercise enhances white matter integrity after ischemic stroke. (A) Schematic diagram of sampling position (dark pink). Created with Procreate 5.3.10. (B) The tMCAO + PE group showed superior preservation of white matter integrity, demonstrated by LFB staining. Dotted lines indicate the corpus callosum region. Scale bar: 100 μm. (C) Quantification of myelin covered area (*n* = 4). (D) The tMCAO + PE group exhibited stronger expression of MBP protein. Dotted lines indicate the corpus callosum region. Scale bar: 100 μm. (E) Quantification of MBP fluorescence intensity (*n* = 8). Data are expressed as mean ± SD. *P < 0.05, **P < 0.01, ***P < 0.001 (one-way analysis of variance followed by Tukey’s honestly significant difference test). (F) A trend toward higher numbers of myelin-covered axons was observed in the tMCAO + PE group relative to the tMCAO group. Scale bar: 1 μm. EC: External capsule; PE: physical exercise; Sham: sham-operated; tMCAO: transient middle cerebral artery occlusion.

To investigate the impact of PE on white matter repair, we used Luxol fast blue staining, MBP immunofluorescence staining, and transmission electron microscopy to examine white matter integrity. White matter tissue of the external capsule and striatum had a compact and clear structure in the Sham group, whereas it was significantly looser and less distinct in the tMCAO group. This suggests that cerebral ischemia injury markedly impaired white matter integrity (**Figure 2A** and **B**). As anticipated, PE significantly promoted white matter repair, shown by an increased myelin-covered area (**Figure 2C**). MBP is a strong alkaline membrane protein produced by oligodendrocytes in the vertebrate central nervous system; it maintains the stability of myelin sheath structure and function. As shown in **Figure 2D** and **E**, PE enhanced MBP expression in both the external capsule and striatum (*P* < 0.05). Regarding the myelin microstructure, we found that tMCAO surgery caused significant thinning of myelin thickness and a notable loss of myelinated axons in the peri-infarct area of the external capsule. In contrast, PE increased myelin sheath thickness, with more myelinated axons observed in the peri-infarct area of the external capsule in tMCAO + PE mice (**Figure 2F**). These findings suggest that PE has beneficial effects on functional recovery and white matter integrity following stroke.

### Physical exercise alleviates neuroinflammatory responses and enhances microglia engulfment of myelin debris

**Figure 3:**
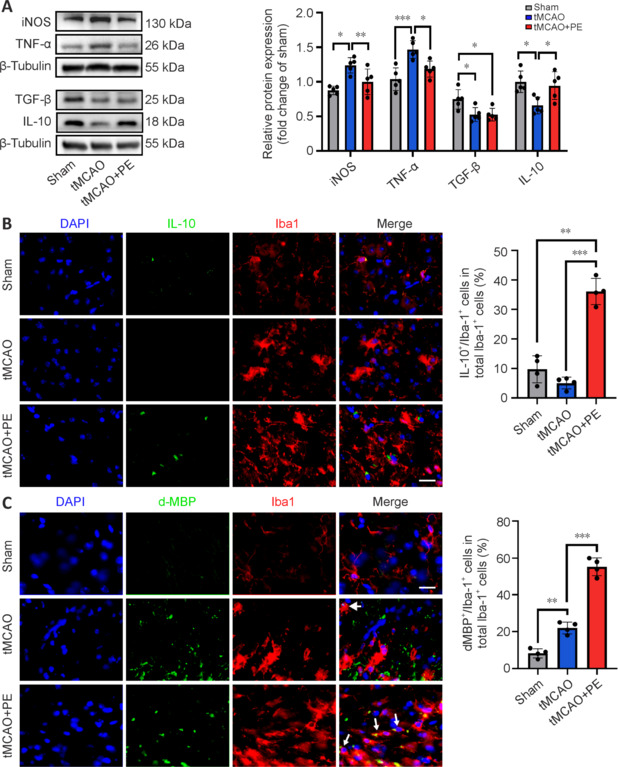
Physical exercise alleviates neuroinflammation and enhances phagocytic activity of microglia after ischemic stroke. (A) Immunoblotting analyses of inflammation-related proteins 21 days after intervention (*n* = 5). (B) More Iba1 (red; Alexa Fluor® 568) co-localized with IL-10 (green; Alexa Fluor® 488) in the tMCAO + PE group (*n* = 4). Scale bar: 20 μm. (C) More Iba1 (red; Alexa Fluor® 568) co-localized with myelin debris (dMBP) (green; Alexa Fluor® 488) in the tMCAO + PE group (*n* = 4). Scale bar: 20 μm. Data are expressed as mean ± SD. *P < 0.05, **P < 0.01, ***P < 0.001 (one-way analysis of variance followed by Tukey’s honestly significant difference test). DAPI: 4′,6-Diamidino-2-phenylindole; dMBP: degraded myelin basic protein; Iba1: ionized calcium-binding adapter molecule 1; IL-10: interleukin 10; iNOS: inducible isoform of nitric oxide synthase; PE: physical exercise; Sham: sham-operated; TGF-β: transforming growth factor-beta; tMCAO: transient middle cerebral artery occlusion; TNF-α: tumor necrosis factor-alpha.

Neuroinflammation is the response of the central nervous system to ischemic brain injury. Excessive and uncontrolled neuroinflammation can hinder white matter repair (Stokowska et al., 2023). In our tMCAO model, we detected several inflammatory mediators in the peri-infarct area, including iNOS, TNF-α, TGF-β, and IL-10. Compared with the Sham group, protein expression levels of proinflammatory factors (iNOS and TNF-α) were markedly increased after tMCAO surgery, whereas anti-inflammatory factors (TGF-β and IL-10) were significantly decreased (**Figure 3A**). However, PE downregulated the protein expression levels of iNOS and TNF-α but upregulated the expression of IL-10. The mRNA expression of these mediators was further confirmed by qPCR (**Additional Figure 1A**). Immunofluorescence staining reinforced these findings showing an anti-inflammatory effect of PE, with an increased number of IL-10-positive microglia in the tMCAO + PE group compared with the tMCAO group (**Figure 3B**).

Demyelination is a common pathological change following stroke (Chen et al., 2020a). The clearance of myelin debris is essential for remyelination (Lampron et al., 2015). As microglia are the primary debris-clearing cells in the central nervous system (Kent and Miron, 2024), this prompted us to examine the impact of PE on microglial phagocytic activity using immunofluorescence staining. **Figure 3C** shows a higher proportion of Iba1^+^ cells contain internalized dMBP^+^ particles (a marker for degraded myelin basic protein (Zhan et al., 2015) in the tMCAO + PE group compared with the tMCAO group, indicating that PE can augment phagocytosis of myelin debris by microglia. Furthermore, mRNA expression of two markers closely associated with phagocytosis and lipid metabolism in microglia, CD68 and triggering receptor expressed on myeloid cells 2 (Trem2), were significantly upregulated in the tMCAO + PE group compared with the tMCAO group (**Additional Figure 1B**). Immunofluorescence staining further indicated that PE upregulated CD68 expression on transmembrane protein 119 (TMEM119)^+^ microglia.

These data suggest that PE can alleviate neuroinflammatory responses and enhance the capacity of microglia to engulf myelin debris, thereby contributing to stroke recovery.

### Treg cells are essential for physical exercise-induced neuroprotection

**Figure 4:**
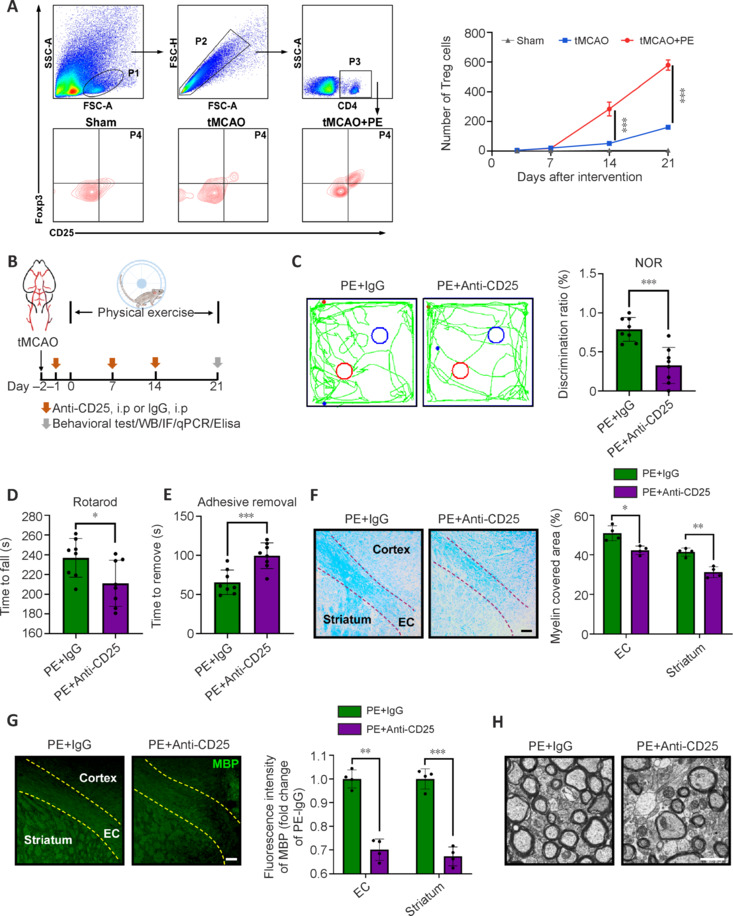
Treg cells are essential for physical exercise-induced functional recovery and white matter repair after ischemic stroke. (A) Flow cytometry analysis of CD4^+^CD25^+^Foxp3^+^ Treg cells in the ischemic brain at 3, 7, 14, and 21 days after intervention (*n* = 3–5). (B) Experimental design for depleting Treg cells. Created with Procreate 5.3.10. (C) The PE + IgG group showed superior recognition of novel objects in the NOR test (*n* = 8). Red circles represent novel objects, and blue circles represent familiar objects. (D, E) Treg cell depletion weakened PE-induced functional recovery determined by the rotarod test (*n* = 8) (D) and adhesive test (*n* = 8) (E). (F) The PE + IgG group showed superior preservation of white matter integrity, demonstrated by LFB staining (*n* = 4). (G) The PE + IgG group showed stronger immunoreactivity of MBP (*n* = 4). Dotted lines indicate the corpus callosum region. Data are expressed as mean ± SD. *P < 0.05, **P < 0.01, ***P < 0.001 (for comparisons among three groups, statistical analysis was performed using one-way analysis of variance followed by Tukey’s honestly significant difference test. For comparisons between two groups, *t*-tests were used). (H) The number of myelin-covered axons in the PE + IgG group was markedly higher than in the tMCAO group. Scale bar: 1 μm. EC: External capsule; Elisa: enzyme-linked immunosorbent assay; Foxp3: forkhead box P3; i.p: intraperitoneal injection; IF: immunofluorescence staining; LFB: Luxol fast blue staining; MBP: myelin basic protein; PE: physical exercise; qPCR: quantitative polymerase chain reaction; Sham: sham-operated; tMCAO: transient middle cerebral artery occlusion; Treg cell: regulatory T cell; WB: Western blot assay.

A previous study demonstrated that PE modulates peripheral immune and tissue homeostasis by enhancing the recruitment of Treg cells (Fernandes et al., 2019). However, the specific role of Treg cells in PE-induced neuroprotection in ischemic brain injury (particularly through an increase their numbers) remains unclear. We first examined the number of Treg cells in the ischemic brain using flow cytometry at 3, 7, 14, and 21 days post-PE. Our results showed that PE increased the number of brain-infiltrating Treg cells at 14 and 21 days but not at 3 and 7 days (**Figure 4A**). To determine the role of Treg cells in PE-induced neuroprotection, we depleted Treg cells by intraperitoneal injection of a CD25-specific antibody (**Figure 4B** and **Additional Figure 2A**). Compared with the PE + IgG group, Treg cell deficiency exacerbated neurological impairments, including cognitive and sensorimotor dysfunction. This was demonstrated by a reduced discrimination ratio in the NOR test (**Figure 4C**), decreased time spent on a rotating bar in the rotarod test (**Figure 4D**), and increased removal time of adhesive tape from impaired paws in the adhesive removal test (**Figure 4E**). These findings underscore the crucial role of Treg cells in PE-mediated functional recovery after stroke. In terms of white matter integrity-related indicators, we observed a reduction in both myelin-covered area and MBP expression levels in the PE + Anti-CD25 group (**Figure 4F** and **G**). Consistent with these findings, electron microscopy showed that more axons without myelin sheaths or with thin myelin sheaths were detected in the PE + Anti-CD25 group (**Figure 4H**).

**Figure 5:**
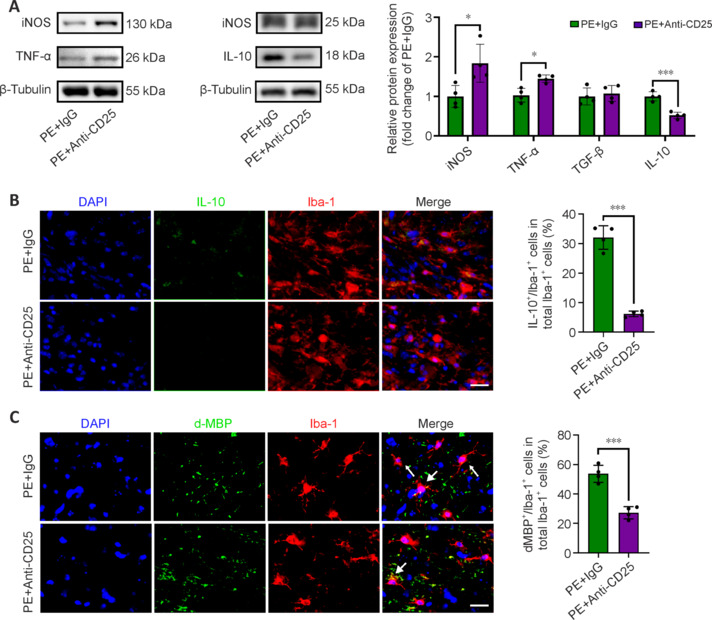
Treg cells are essential for physical exercise-induced reduction of inflammation and promotion of phagocytosis after ischemic stroke. (A) Immunoblotting analyses of inflammation-related proteins 21 days after intervention (*n* = 4). (B) More Iba1 (red; Alexa Fluor® 568) co-localized with IL-10 (green; Alexa Fluor® 488) in the PE + IgG group (*n* = 4). Scale bar: 20 μm. (C) More Iba1 (red; Alexa Fluor® 568) co-localized with myelin debris (dMBP) (green; Alexa Fluor® 488) in the PE + IgG group (*n* = 4). Scale bar: 20 μm. Data are expressed as mean ± SD. *P < 0.05, **P < 0.01, ***P < 0.001 (unpaired *t*-test). DAPI: 4′,6-Diamidino-2-phenylindole; dMBP: degraded myelin basic protein; Iba1: ionized calcium-binding adapter molecule 1; IL-10: interleukin 10; iNOS: inducible isoform of nitric oxide synthase; PE: physical exercise; TGF-β: transforming growth factor-beta; TNF-α: tumor necrosis factor-alpha; Treg cell: regulatory T cell.

To gain detailed insight into the role of Treg cells in neuroinflammation and phagocytosis, we examined inflammatory markers and myelin debris engulfment in mice after Treg cell depletion on day 21 post-PE. Compared with the PE + IgG group, the PE + Anti-CD25 group exhibited a significant increase in the expression of pro-inflammatory markers, such as iNOS and TNF-α, whereas the expression of the anti-inflammatory marker, IL-10, was markedly reduced (**Figure 5A**, **B** and **Additional Figure 2B**). Furthermore, Treg cell depletion attenuated the PE-induced phagocytic effect, shown by a reduced proportion of Iba1^+^ cells containing internalized dMBP^+^ particles (**Figure 5C**) and decreased expression of CD68 and Trem2 (**Additional Figure 2C**).

These findings suggest that Treg cells are crucial for PE-induced neuroprotection, potentially through modulation of microglia function.

### Osteopontin may be the molecular bridge for Treg cells to regulate microglial function

**Figure 6:**
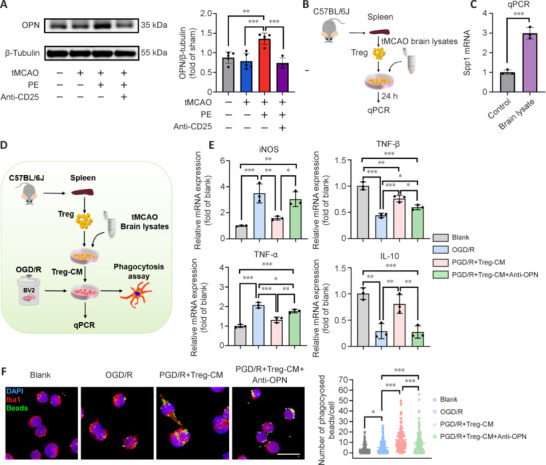
Osteopontin may be the molecular bridge for Treg regulation of microglial function after ischemic stroke. (A) Immunoblotting analyses of OPN 21 days after intervention (*n* = 4–5). (B) Experimental design for mRNA extraction from Treg cells. (C) mRNA expression of Spp1 in Treg cells. (D) Experimental design for BV2 microglia *in vitro*. Created with Microsoft PowerPoint 2019. (E) Expression of inflammation-related genes. (F) More fluorescent microbeads (green) co-localized with microglia (Iba1^+^) (red; Alexa Fluor® 555). Scale bar: 50 μm. Data are expressed as mean ± SD. *P < 0.05, **P < 0.01, ***P < 0.001 (one-way analysis of variance followed by Tukey’s honestly significant difference test). CM: Conditioned medium; DAPI: 4′,6-diamidino-2-phenylindole; Iba1: ionized calcium-binding adapter molecule 1; IL-10: interleukin 10; iNOS: inducible isoform of nitric oxide synthase; OGD/R: oxygen glucose deprivation/re-oxygenation; OPN: osteopontin; PE: physical exercise; qPCR: quantitative polymerase chain reaction; TGF-β: transforming growth factor-beta; tMCAO: transient middle cerebral artery occlusion; TNF-α: tumor necrosis factor-alpha; Treg cell: regulatory T cell.

It is reported that the interplay between Treg cells and microglia mediated by OPN is crucial for white matter repair (Shi et al., 2021). Furthermore, the exogenously synthesized OPN-peptide, icosamer, has anti-inflammatory properties and can enhance microglial phagocytic activity *in vitro* (Kim et al., 2017; Lee et al., 2018). Initially, we detected OPN protein expression in the peri-infarct area. **Figure 6A** shows that PE significantly upregulated OPN expression, whereas OPN was significantly inhibited by Treg depletion. Subsequently, we examined the expression of secreted phosphoprotein 1 (Spp1; the gene encoding OPN) on isolated Treg cells. **Figure 6B** and **C** shows that compared with the control group, mRNA expression of Spp1 in Treg cells was markedly upregulated after stimulation with ischemic brain lysates, consistent with a previous study (Shi et al., 2021).

To further validate the interaction between Treg cells and microglia mediated by OPN, we established an *in vitro* oxygen–glucose deprivation and reperfusion (OGD/R) model using BV2 microglia. **Figure 6D** and **E** shows that OGD/R significantly induced the expression of iNOS and TNF-α, while suppressing the expression of TGF-β and IL-10. To mimic the augmentation of Treg cells observed in the animal model, BV2 cells were treated with 10% Treg-CM for 18 hours, starting at 6 hours after reperfusion. At 24 hours post-reperfusion, Treg-CM markedly reduced pro-inflammatory factors and enhanced anti-inflammatory factors. However, the anti-inflammatory effects of Treg-CM were significantly attenuated by neutralization of OPN using an anti-OPN antibody.

To determine the effect of OPN on microglial phagocytosis modulated by Treg cells, microglia were incubated with fluorescent microbeads for 1 hour at 37°C (**Figure 6F**). In the Blank group, untreated microglia exhibited a very low level of phagocytic activity, engulfing only 3.71 ± 4.23 beads per cell. However, OGD/R treatment significantly enhanced phagocytic activity, increasing the bead count to 5.48 ± 4.89 per cell (*P* < 0.05). Furthermore, exposure to Treg-CM further potentiated the phagocytic activity of microglia, with an average of 12.80 ± 10.40 beads per cell, and some cells engulfing up to 50 microbeads. As anticipated, anti-OPN antibody mitigated the effect of Treg-CM. Additionally, we found that Treg-BV2-CM attenuated OGD/R-induced oligodendrocyte death and promoted MBP expression (**Additional Figure 3**).

**Figure 7:**
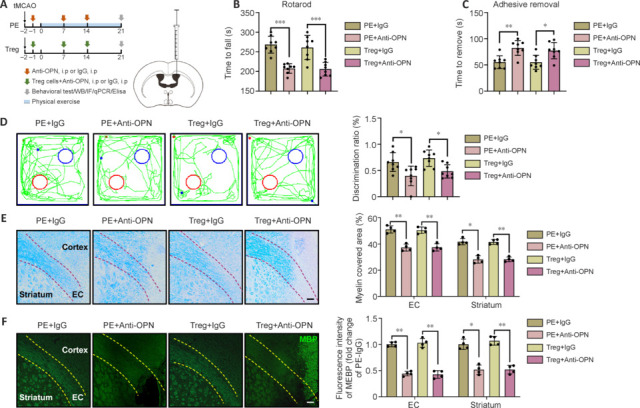
Osteopontin blockade weakens physical exercise or Treg cell induced functional recovery and white matter repair. (A) Experimental design for OPN blockade. Created with Procreate 5.3.10. (B, C) OPN blockade weakened PE or Treg cell induced functional recovery determined by the rotarod test (*n* = 8) (B) or adhesive test (*n* = 8) (C). (D) The PE + IgG and Treg + IgG groups showed superior recognition of novel objects in the NOR test (*n* = 8). Red circles represent novel objects, and blue circles represent familiar objects. (E, F) OPN blockade dampened PE or Treg cell induced white matter repair determined by LFB straining (*n* = 4) (E) and MBP (green; Alexa Fluor® 488) immunostaining (*n* = 4) (F) 21 days after intervention. Dotted lines indicate the corpus callosum region. Data are expressed as mean ± SD. *P < 0.05, **P < 0.01, ***P < 0.001 (one-way analysis of variance followed by Tukey’s honestly significant difference test). EC: External capsule; i.p: intraperitoneal injection; LFB: Luxol fast blue staining; MBP: myelin basic protein; NOR: novel object recognition; OPN: osteopontin; PE: physical exercise; tMCAO: transient middle cerebral artery occlusion; Treg cell: regulatory T cell.

To further verify that OPN plays a pivotal role in Treg cell-mediated neuroprotection, we conducted an *in vivo* experiment using OPN neutralizing antibodies. We found that direct infusion of Treg cells had a comparable effect to PE, thereby supporting the hypothesis that PE exerts neuroprotective effects by augmenting Treg cells. However, OPN blockade exacerbated the neurological deficits (**Figure 7A–D** and **Additional Figure 4**) and hindered white matter repair (**Figure 7E** and **F**), resembling the effects of Treg cell depletion.

These results suggest that Treg cells, elevated by PE, likely modulate microglial function through OPN secretion, ultimately contributing to white matter repair.

### Physical exercise boosts brain Treg cells via CXCL12 upregulation

**Figure 8:**
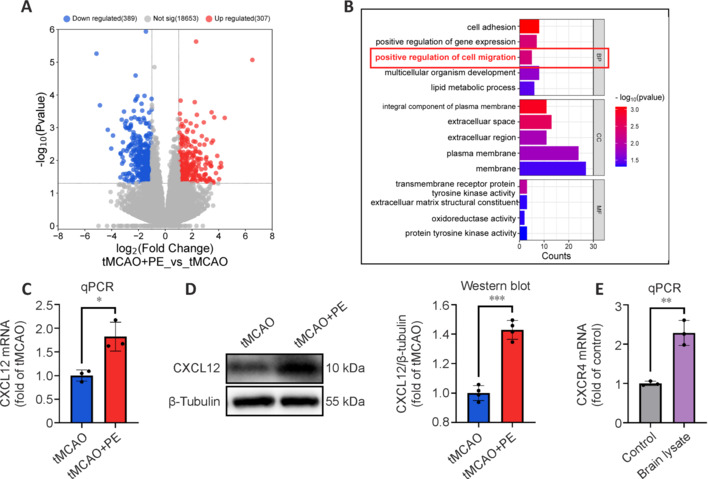
CXCL12 may drive Treg cell infiltration into the brain after ischemic stroke. (A) Volcano plots of DEGs. Red and blue represent upregulation and downregulation, respectively. (B) The top five terms of biological processes, molecular functions, and cellular components from GO analysis of DEGs. (C) mRNA expression of CXCL12 in the peri-infarct area 21 days after intervention (*n* = 3). (D) Immunoblotting analyses of CXCL12 in the peri-infarct area 21 days after intervention (*n* = 4). (E) mRNA expression of CXCR4 in Treg cells. Data are expressed as mean ± SD. *P < 0.05, **P < 0.01, ***P < 0.001 (unpaired *t*-tests). BP: Biological processes; CC: cellular components; CXCL12: C–X–C motif chemokine ligand 12; DEG: differentially expressed gene; GO: Gene Ontology; MF: molecular functions; PE: physical exercise; qPCR: quantitative polymerase chain reaction; tMCAO: transient middle cerebral artery occlusion; Treg cell: regulatory T cell.

The migration of Treg cells towards the peri-infarct area depends on the chemokines released by injured tissue and their corresponding chemokine receptors (Cai et al., 2022). To identify the specific chemokines that facilitate the infiltration of Treg cells into the brain, we conducted RNA sequencing. Compared with the tMCAO group, the tMCAO + PE group exhibited 696 differentially expressed genes, 307 upregulated and 389 downregulated (**Figure 8A**). Subsequently, we performed GO enrichment analysis of these differentially expressed genes and found enrichment of the term “positive regulation of cell migration” encompassing: CXCL12, PODXL, FLT4, ITGAX, and SEMA3G (**Figure 8B**). Given that CXCL12 is known to induce Treg cell migration via C–X–C motif chemokine receptor 4 (CXCR4), we further investigated CXCL12 expression in the brain. Both mRNA and protein levels of CXCL12 were significantly increased in the brains of tMCAO + PE mice compared with tMCAO mice (**Figure 8C** and **D**). Additionally, mRNA expression of CXCR4 on Treg cells was notably upregulated after stimulation with ischemic brain lysates (**Figure 8E**). These results demonstrate that upregulation of CXCL12 may enhance the migration of Treg cells to the ischemic brain following PE.

## Discussion

Our study fills a gap between the involvement of Treg cells and PE for stroke rehabilitation. We show that Treg cells are essential for the neuroprotective effects of PE on ischemic injured mice, especially for PE-induced white matter repair. PE increased the number of brain-infiltrating Treg cells in the tMCAO mouse model, while Treg cell deficiency weakened the beneficial effects of PE on neurological function recovery and white matter repair. Besides their reported anti-inflammatory effects, Treg cells further enhanced the phagocytotic activity of microglia to clear myelin debris via expression of OPN. This boosting effect of PE on Treg cells in the brain may be mediated by upregulated expression of the chemokine CXCL12.

PE is a widely adopted rehabilitation method for stroke that significantly contributes to neurological function (Zheng et al., 2025). Numerous preclinical and clinical studies have demonstrated its neuroprotective effects, which are linked to inhibition of oxidative stress (Toborek et al., 2013), enhancement of angiogenesis (Gertz et al., 2006), neuro-regeneration (Wu et al., 2022), and suppression of glia responses (Xu et al., 2023). However, the underlying molecular mechanisms remain elusive. Due to breakdown of the blood–brain barrier, peripheral immune cells, including T lymphocytes, infiltrate the injured brain and influence its repair. Although T cells are reported to aggravate ischemic brain injury, emerging evidence supports the beneficial effect of Treg cells on brain repair (Ito et al., 2019; Yshii et al., 2022), especially on white matter repair (Shi et al., 2021). Long-term neurological recovery after ischemic injury is strongly related to the repair and functional restoration of injured white matter (Pu et al., 2021). Treg cell depletion aggravates white matter lesions and exacerbates long-term functional recovery 21 days after tMCAO, while increased IL-2/IL-2 antibody-mediated Treg improves white matter integrity and behavioral outcomes (Shi et al., 2021; Yuan et al., 2023). A recent study highlighted exercise as a natural Treg booster (Langston et al., 2023), especially under pathological conditions. For example, in an experimental autoimmune encephalomyelitis model, PE reduced disease severity and neuropathology scores by elevating Treg cells (Chen et al., 2021). However, the impact of PE on Treg cells in the ischemic stroke model is still unknown. Our study found that PE significantly increased the number of Treg cells in ischemic brain at 14 and 21 days post-intervention, rather than 3 and 7 days. This suggests that a certain duration of PE is required to trigger augmentation of Treg cells, consistent with the gradual nature of clinical recovery. Crucially, our results showed that depletion of Treg cells via intraperitoneal administration of CD25-specific antibody diminished PE-mediated improvement in neurological function. Meanwhile, adoptive transfer of Treg cells to tMCAO mice produced a similar effect to PE, confirming the positive impact of Treg cells. Most studies also support a neuroprotective role of Treg cells, including suppression of neurotoxic astrogliosis (Ito et al., 2019) and neuroinflammation (Yshii et al., 2022). Controversially, Kleinschnitz et al. (2013) proposed that adoptive transfer of Treg cells exacerbated brain injury in tMCAO mice by inducing microvascular dysfunction. The discrepancy between our data and Kleinschnitz’s study may be attributed to the timing of Treg cell augmentation: occurring before or after tMCAO surgery. In our study, PE induced a delayed increase in Treg cells post-tMCAO. While Kleinschnitz et al. (2013) used adoptive transfer of Treg cells 24 hours prior to tMCAO, it is noteworthy that Treg depletion prior to or at late stage of inflammation exhibits different effects (Hu et al., 2013; Liu et al., 2017). In addition, most studies delivering Treg cells after tMCAO suggest beneficial effects. These data suggest that Treg cells are essential for PE-induced neural repair.

Microglia are the resident immune cells of the central nervous system, and are highly heterogeneous under pathological conditions (Li et al., 2019; Hasel et al., 2023; Chen et al., 2025). Ischemic injury can induce activation of microglia, leading to the release of pro-inflammatory cytokines that contribute to white matter damage (Zhang et al., 2020b). While on the other hand, microglia facilitate white matter repair by clearing cellular debris (Zheng et al., 2022; Cao et al., 2023) and creating a favorable environment for healing. The microglial phenotype that exhibits reparative properties is primarily determined by their surrounding environment and interactions with other cells (Zhang et al., 2024). As previously reported (Shi et al., 2021), Treg cells alter microglia function. Primarily, we observed that the presence of Treg cells significantly reduces the inflammatory levels of microglia, both *in vivo* and *in vitro*. Furthermore, we confirmed that Treg cells can enhance the phagocytic capacity of microglia, promoting their ability to phagocytose myelin debris, which is crucial for white matter repair (Lampron et al., 2015). A previous study found that astrocytes phagocytose microglial debris to remove dead microglia (Zhou et al., 2022). In addition, endothelial cells can engulf myelin debris and this plays an important role in promoting fibrotic scar formation in demyelination disorders (Zhou et al., 2022). Although other cells in the brain also have phagocytic functions, the Treg cell–microglia interplay may play a more positive role in promoting white matter repair. The influence of Treg cells on other cell types and its implications remain to be further investigated.

OPN is reported as a key mediator of the Treg cell–microglia interaction (Shi et al., 2021). A previous study showed that OPN is expressed by Treg cells. Compared with circulating Treg cells, a higher percentage of brain infiltrating Treg cells expressed OPN (Shi et al., 2021). Consistent with this study, we found that expression of Spp1 (the gene encoding OPN) on Treg cells was significantly increased after stimulation by ischemic brain lysates. In addition, PE increased expression of OPN, while depletion of Treg cells weakened this effect. These results indicate that Treg cells are an important cellular source of OPN, but not the only one. Peri-infarct microglia begin to express OPN as early as 24 hours after stroke (Ellison et al., 1998). In a brain ischemic injury model, OPN has been implicated in different features of stroke pathology, including alleviating neuroinflammation (Zhang et al., 2021), protecting the blood–brain barrier (Gliem et al., 2015), inhibiting neuronal apoptosis (Topkoru et al., 2013), and mediating survival, proliferation, and migration of neural stem cells (Rabenstein et al., 2015). In the present study, we found that anti-OPN antibody can reduce the effects of Treg-CM on anti-inflammatory and phagocytosis promotion, suggesting an important role of OPN in regulating the Treg–microglia interplay. Also, Treg cells are a possible trigger for OPN production in microglia (Shi et al., 2021), but the mechanism remains to be explored.

Our study showed that PE increased the population of Treg cells in both the brain and spleen. Augmentation of peripheral immune cells induced by PE are likely due to sympathetic nervous system activation and metabolic regulation (Dorneles et al., 2020). However, their migration towards injury sites relies on the expression of chemokines and their receptors. Consistent with a previous study (Luo et al., 2014), we also observed upregulation of CXCL12, a pivotal Treg cell chemokine, at both the mRNA and protein levels post-PE. Multiple chemokines, including CCL1 (Ito et al., 2019), CCL2 (Zhu et al., 2023), CCL5 (Li et al., 2017), CXCL14 (Lee et al., 2017), and CCL20 (Ito et al., 2019), can reportedly recruit Treg cells into the brain, yet our sequencing data showed no upregulation of these chemokines in response to PE. Hypoxia-inducible factor-1α serves as a crucial transcription factor regulating CXCL12 (Ceradini et al., 2004), and its expression can be activated by exercise (Wu et al., 2020). Our finding suggests that PE enhanced the production of CXCL12 in the brain, potentially linked to PE-induced relative hypoxia activating hypoxia-inducible factor-1α expression, and thereby enhancing CXCL12 transcription. However, robust data are needed to validate the underlying mechanisms.

Several aspects of our present study need to be considered. First, we focused on the critical role of Treg cell expansion in the central nervous system for stroke recovery. Although our results indicate that PE can also reduce the expression of peripheral proinflammatory cytokines (**Additional Figure 5**), we did not further investigate the effects of PE on peripheral immune cells. These will be examined in future research. Second, in our *in vivo* experiments, we used antibodies to neutralize OPN in the brain without specifically knocking down OPN expression in Treg cells. Consequently, we can only provide indirect evidence that OPN acts as a significant mediator in the interaction between Treg cells and microglia. Direct evidence for this interaction comes solely from *in vitro* data. Then, although oligodendrocyte proliferation and differentiation are not the focus of our study, they are crucial for white matter repair. Additionally, Treg cells are reported to directly facilitate myelin regeneration in mouse models of multiple sclerosis. Lastly, this study was only conducted on male mice, partly because we found that most studies on stroke followed this approach, and partly because we wanted to minimize the interference of confounding factors, given the increasing reports on the impact of sexual dimorphism on Treg cell function (Tejera-Alhambra et al., 2012; Dimitrijević et al., 2019).

In summary, our study demonstrates that PE boosts Treg cells in the brain and periphery post-stroke. These cells are crucial for PE-mediated white matter repair and stroke recovery, likely due to the regulatory effects of Treg cells on microglial function, and specifically by enhancing their anti-inflammatory phenotype and myelin debris clearance capabilities. In addition, PE may elevate CXCL12 to recruit Treg cells into the brain. This study further highlights the mechanisms by which exercise modulates the immune system to contribute to stroke recovery, presenting novel intervention targets. This research deepens our understanding of the role of PE in immune modulation during stroke recovery, and offers new targets for intervention.

## Additional files:

***Additional Table 1:*** *Primer sequences.*

***Additional Figure 1:*** *PE alleviates neuroinflammation and enhances phagocytic activity of microglia after ischemic stroke.*

Additional Figure 1 PE alleviates neuroinflammation and enhances phagocytic activity of microglia after ischemic stroke.
(A) Expression of inflammation-related genes in the Sham, tMCAO and tMCAO + PE groups 21 days after intervention (*n* = 3). (B) Expression of mRNA for phagocytosis-related genes in the Sham, tMCAO, and tMCAO + PE groups 21 days after intervention (*n* = 3). (C) More microglia (TMEM119+) (green; Alexa Fluor® 488) co-localized with CD68 (green; Alexa Fluor® 568) in the tMCAO + PE group. Scale bar: 20 μm. Data are expressed as mean ± SD. ^*^P < 0.05, ^**^P < 0.01, ^***^P < 0.001 (one-way analysis of variance followed by Tukey’s honestly significant difference test). DAPI: 4',6-Diamidino-2-phenylindole; IL-10: interleukin 10; iNOS: inducible isoform of nitric oxide synthase; PE: physical exercise; Sham: sham-operated; TGF-β: transforming growth factor-beta; tMCAO: transient middle cerebral artery occlusion; TMEM119: transmembrane protein 119; TNF-α: tumor necrosis factor-alpha; Trem2: triggering receptor expressed on myeloid cells 2.

***Additional Figure 2:*** *Treg cell depletion attenuates PE-mediated protection.*

Additional Figure 2 Treg cell depletion attenuates PE-mediated protection.
(A) Quantitative evaluation of Treg cell depletion efficiency (*n* = 3). (B) Expression of inflammation-related genes in PE + IgG and PE + Anti-CD25 groups 21 days after intervention (*n* = 3). (C) Expression of phagocytosis-related genes in PE + IgG and PE + Anti-CD25 groups 21 days after intervention (*n* = 3). Data are expressed as mean ± SD. ^*^P < 0.05, ^**^P < 0.01, ^***^P < 0.001 (unpaired *t*-test). Foxp3: Forkhead box P3; IL-10: interleukin 10; iNOS: inducible isoform of nitric oxide synthase; PE: physical exercise; TGF-β: transforming growth factor-beta; TNF-α: tumor necrosis factor-alpha; Trem2: triggering receptor expressed on myeloid cells 2.

***Additional Figure 3:*** *Treg-BV2 interplay is beneficial for the survival of MO3.13 oligodendrocytes.*

Additional Figure 3 Treg-BV2 interplay is beneficial for the survival of MO3.13 oligodendrocytes.
(A) Oligodendrocyte viability was assessed by MTT. (B) Immunoblotting analyses of MBP. Data are expressed as mean ± SD. ^*^P < 0.05, ^**^P < 0.01, ^***^P < 0.001 (one-way analysis of variance followed by Tukey’s honestly significant difference test for post hoc comparisons). CM: Conditioned medium; MBP: myelin basic protein; MTT: 3-(4,5-dimethylthiazolyl-2)-2,5-diphenyltetrazolium bromide; OGD/R: oxygen glucose deprivation/re-oxygenation; Treg: regulatory T cell.

***Additional Figure 4:*** *The blocking efficiency of OPN.*

Additional Figure 4 Blocking efficiency of OPN.
Data are expressed as mean ± SD. ^*^P < 0.05, ^**^P < 0.01, ^***^P < 0.001 (one-way analysis of variance, followed by Tukey’s honestly significant difference test). OPN: Osteopontin; PE: physical exercise; Treg: regulatory T cell.

***Additional Figure 5:*** *Expression of inflammatory factors in peripheral blood.*

Additional Figure 5 Expression of inflammatory factors in peripheral blood.
(A–D) Expression of TNF-α (A), IFN-γ (B), TGF-β (C), and IL-10 (D) in peripheral blood 21 days after intervention (*n* = 3). Data are expressed as mean ± SD. ^*^P < 0.05, ^**^P < 0.01, ^***^P < 0.001 (one-way analysis of variance, followed by Tukey’s honestly significant difference test). IFN-γ: Interferon-gamma; IL-10: interleukin 10; PE: physical exercise; Sham: sham-operated; TGF-β: transforming growth factor-beta; tMCAO: transient middle cerebral artery occlusion; TNF-α: tumor necrosis factor-alpha.

## Data Availability

*All data relevant to the study are included in the article or uploaded as Additional files*.
