# Supplementary figures and images for: Physical exercise promotes white matter repair after ischemic stroke
Source: Neural Regen Res. 2025 Apr 29;21(6):2397–406. doi: 10.4103/NRR.NRR-D-24-00861 (PMC13211827; doi:10.4103/NRR.NRR-D-24-00861)

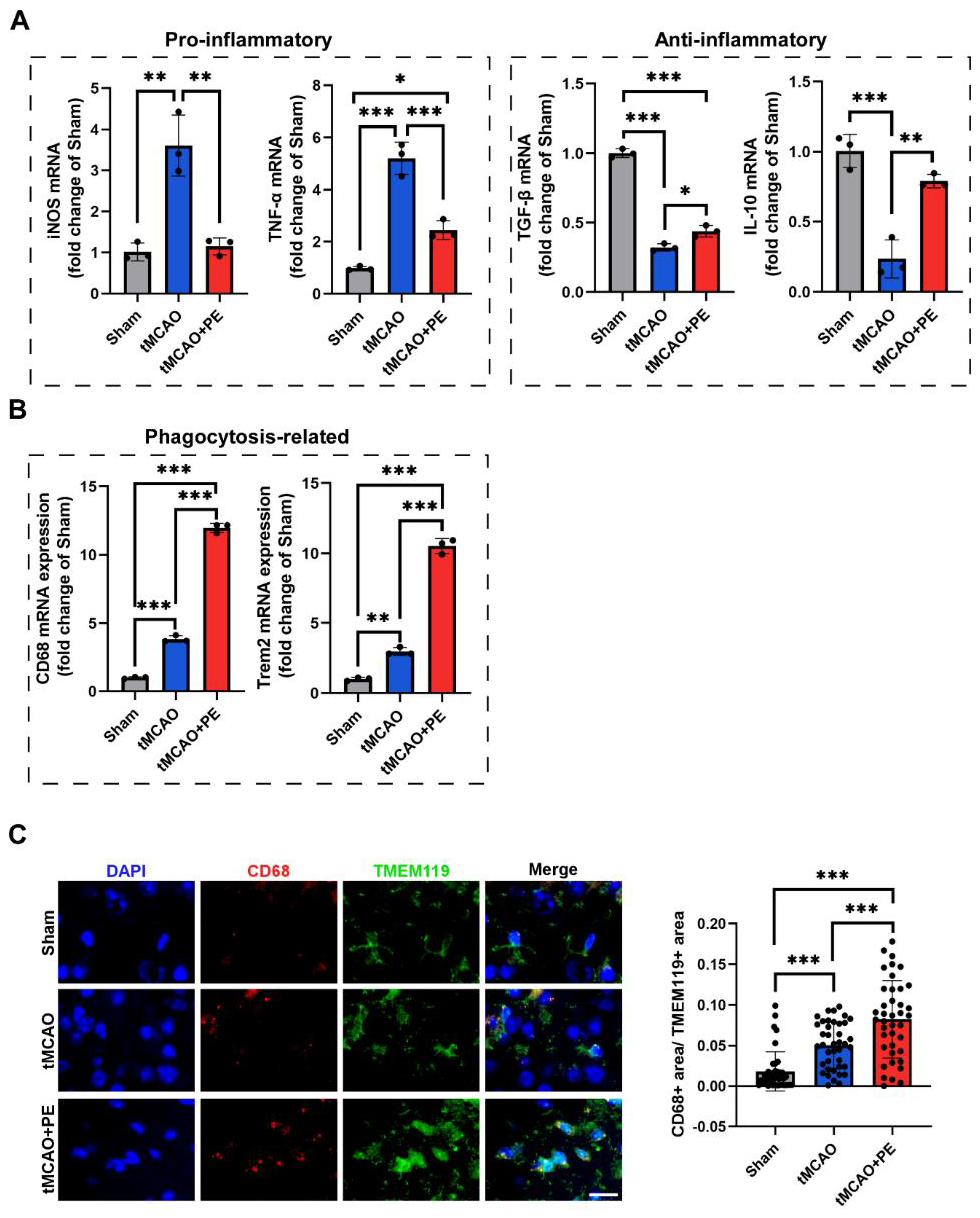

Supplement: Supplementary file 1 [file NRR-21-2397_Suppl1.tif]

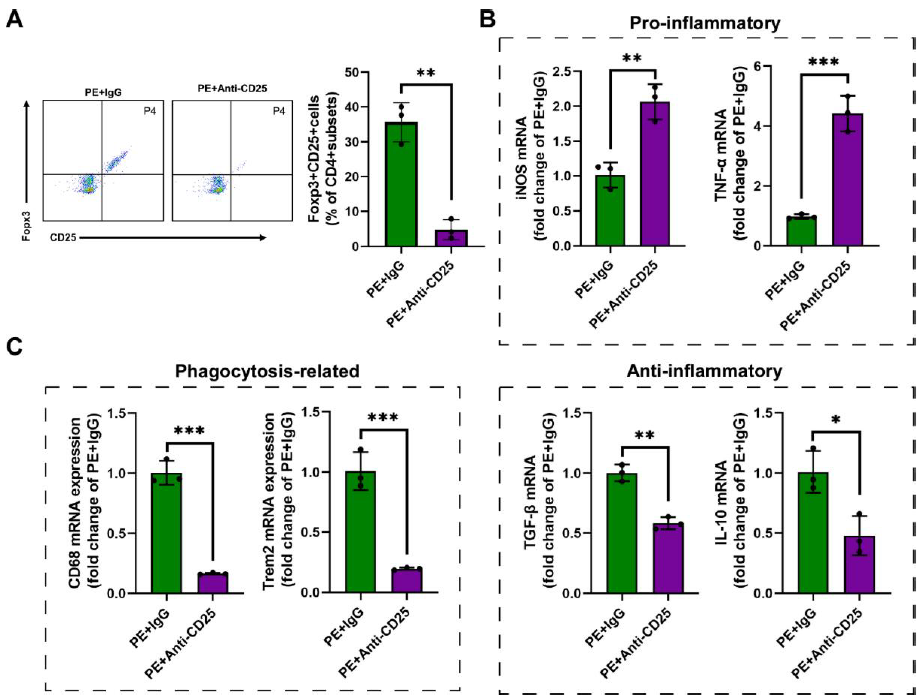

Supplement: Supplementary file 2 [file NRR-21-2397_Suppl2.tif]

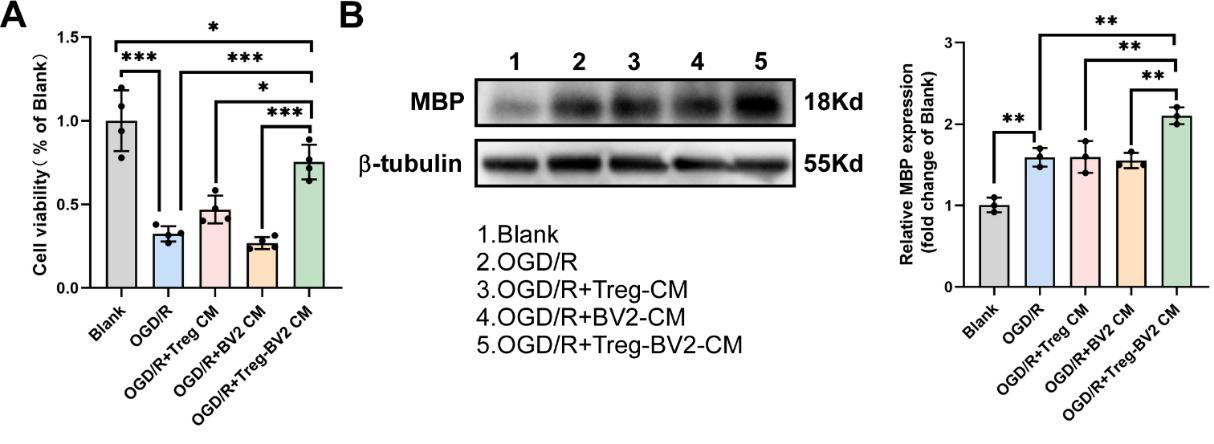

Supplement: Supplementary file 3 [file NRR-21-2397_Suppl3.tif]

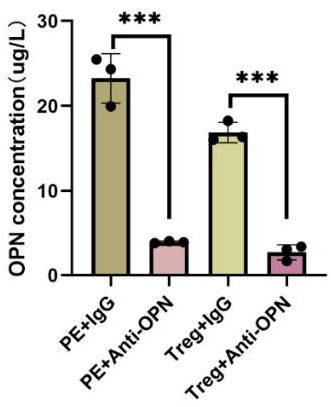

Supplement: Supplementary file 4 [file NRR-21-2397_Suppl4.tif]

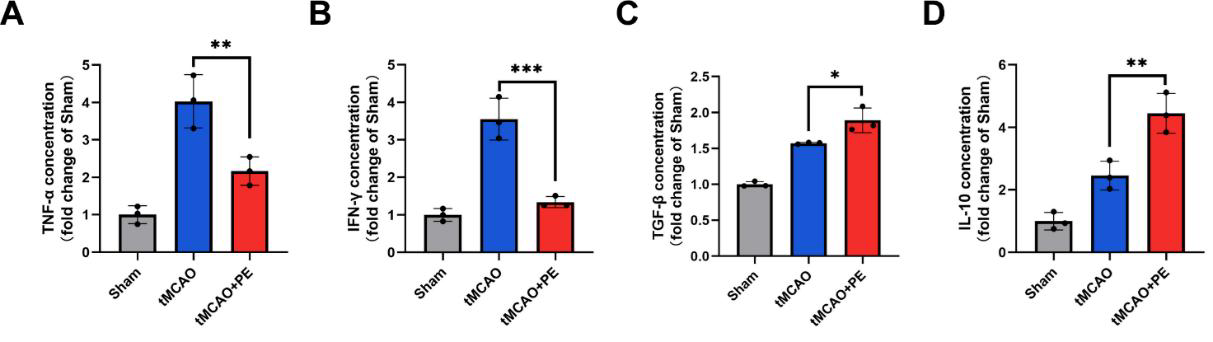

Supplement: Supplementary file 5 [file NRR-21-2397_Suppl5.tif]
